# Editorial Notes

**Published:** 1895-01

**Authors:** 


### Editorial Notes.

---

THE prescription containing quinine, in Dr. Porter's article on "Dengue," in the December issue, should have been ʒiv. *instead of* ℥iv.

---

THERE are 18,484 medical students attending the colleges in the United States and Canada; and still it is comparatively rare that we hear of a physician's death.

---

IT is announced that Dr. William Osler, of the Johns Hopkins University, Baltimore, has been called to the presidency of the McGill University. Dr. Osler is a Canadian by birth and early training, and it will be but a rendering back of her own to Canada if he accepts this new appointment. While we recognize this, we shall none the less regret the loss to the profession in the United States.

---

THE JEFFERSON MEDICAL COLLEGE OF PHILADELPHIA.—Many members of the class of 1879 are desirous of having a class reunion on the occasion of the fifteenth anniversary of their graduation. Owing to changes, comparatively few addresses are known, and, therefore, this means is resorted to with the hope that every member of the class of 1879 who reads this notice will communicate at once with the class president, Dr. Philip R. Koons, Mechanicsburg, Cumberland County, Pennsylvania.

